# Referring heroin users from compulsory detoxification centers to community methadone maintenance treatment: a comparison of three models

**DOI:** 10.1186/1471-2458-13-747

**Published:** 2013-08-13

**Authors:** Liping Yan, Enwu Liu, Jennifer M McGoogan, Song Duan, Li-Tzy Wu, Scott Comulada, Zunyou Wu

**Affiliations:** 1 National Center for AIDS/STD Control and Prevention, Chinese Center for Disease Control and Prevention, Beijing, China; 2 Beijing Haidian District Health School, Beijing, China; 3 Dehong Prefecture Center for Disease Control and Prevention, Yunnan Province, China; 4 Department of Psychiatry and Behavioral Science, Duke University Medical Center, Durham, NC 27710, USA; 5 UCLA Center for Community Health, Los Angeles, CA 90024, USA

**Keywords:** Heroin addiction, Detoxification, Methadone maintenance treatment, HIV, China

## Abstract

**Background:**

Both compulsory detoxification treatment and community-based methadone maintenance treatment (MMT) exist for heroin addicts in China. We aim to examine the effectiveness of three intervention models for referring heroin addicts released from compulsory detoxification centers to community methadone maintenance treatment (MMT) clinics in Dehong prefecture, Yunnan province, China.

**Methods:**

Using a quasi-experimental study design, three different referral models were assigned to four detoxification centers. Heroin addicts were enrolled based on their fulfillment to eligibility criteria and provision of informed consent. Two months prior to their release, information on demographic characteristics, history of heroin use, and prior participation in intervention programs was collected via a survey, and blood samples were obtained for HIV testing. All subjects were followed for six months after release from detoxification centers. Multi-level logistic regression analysis was used to examine factors predicting successful referrals to MMT clinics.

**Results:**

Of the 226 participants who were released and followed, 9.7% were successfully referred to MMT(16.2% of HIV-positive participants and 7.0% of HIV-negative participants). A higher proportion of successful referrals was observed among participants who received both referral cards and MMT treatment while still in detoxification centers (25.8%) as compared to those who received both referral cards and police-assisted MMT enrollment (5.4%) and those who received referral cards only (0%). Furthermore, those who received referral cards and MMT treatment while still in detoxification had increased odds of successful referral to an MMT clinic (adjusted OR = 1.2, CI = 1.1-1.3). Having participated in an MMT program prior to detention (OR = 1.5, CI = 1.3-1.6) was the only baseline covariate associated with increased odds of successful referral.

**Conclusion:**

Findings suggest that providing MMT within detoxification centers promotes successful referral of heroin addicts to community-based MMT upon their release.

## Background

Heroin use and heroin-related HIV infections are important public health concerns in China, and heroin users represent a high-risk population critical to broader HIV infection prevention efforts [1,2]. There are approximately 1.54 million illicit drug users registered by the China’s Ministry of Public Security, the majority of whom (69%) are heroin users [3]. In 1987, the first heroin detoxification centers were established in in Lanzhou city, Gansu province in southwestern China, where illicit drug use is relatively common. As of 2010, approximately 216,000 drug users were detained in detoxification centers across the nation [3]. Drug users sentenced to a compulsory detoxification center are typically detained for up to 2 years. However, drug addiction, particularly heroin addiction, is a chronic condition with a high rate of relapse [4,5]. In China, more than 90% of heroin users relapse after being released from detoxification centers [6-9]. Therefore, it is critical that effective interventions are identified to engage heroin users being released from compulsory detoxification in therapy regimens, such as methadone maintenance treatment (MMT), in order to facilitate recovery.

Methadone treatment (either detoxification or maintenance) has been the primary therapy for opiate addiction in the United States [10]. Studies have demonstrated that methadone treatment is clinically effective as well as cost effective [11,12]. Specifically, MMT is effective at decreasing heroin use, drug-related criminal behaviors, HIV-related risk behaviors, HIV and hepatitis virus transmissions, and mortality among heroin addicts [13-15]. One study has shown that the probability of having an HIV infection was approximately 6 times greater among heroin addicts who had never received MMT as compared to heroin addicts in an MMT program [16]. In response to public health concerns about heroin use and heroin use-related HIV transmission, China’s Ministry of Health launched a national MMT program to provide methadone treatment to heroin addicts in communities [2,16,17]. In 2004, the first eight MMT clinics were opened in the southwestern China, and the program has since expanded to 748 clinics in 2012. However, there are yet many barriers to treatment faced by heroin users, thus the benefits of China’s national MMT program will not be fully realized unless effective means are identified to promote engagement in treatment, and therefore, expansion of coverage.

China’s two separate, and very different, methods for addressing the illicit drug use problem—detoxification in detention centers governed by the Ministry of Public Security and MMT in community clinics administered by the Ministry of Health—highlight a serious gap in the broader national strategies for controlling the drug use and HIV/AIDS epidemics. Implementation of an effective intervention to help transition heroin users released from compulsory detoxification centers to community-based MMT clinics, thereby preventing their relapse and promoting their rehabilitation, is urgently needed. However, strategies for facilitating successful transition from detoxification center to treatment clinic have yet to be studied in China.

It is within this context that we present the current study, which examines the effectiveness of three different referral models to determine whether any of them have a positive impact on rates of successful referral to MMT within six months of release from detoxification centers. The three models examined are: referral cards plus police-assistive MMT enrollment (Model 1), referral cards plus 2 months of MMT in detoxification (Model 2), and referral cards only (Model 3). We hypothesize that treating incarcerated heroin users with methadone prior to their release will promote their enrollment and participation in MMT after their release.

## Methods

### Study design and study sites

**Figure 1:**
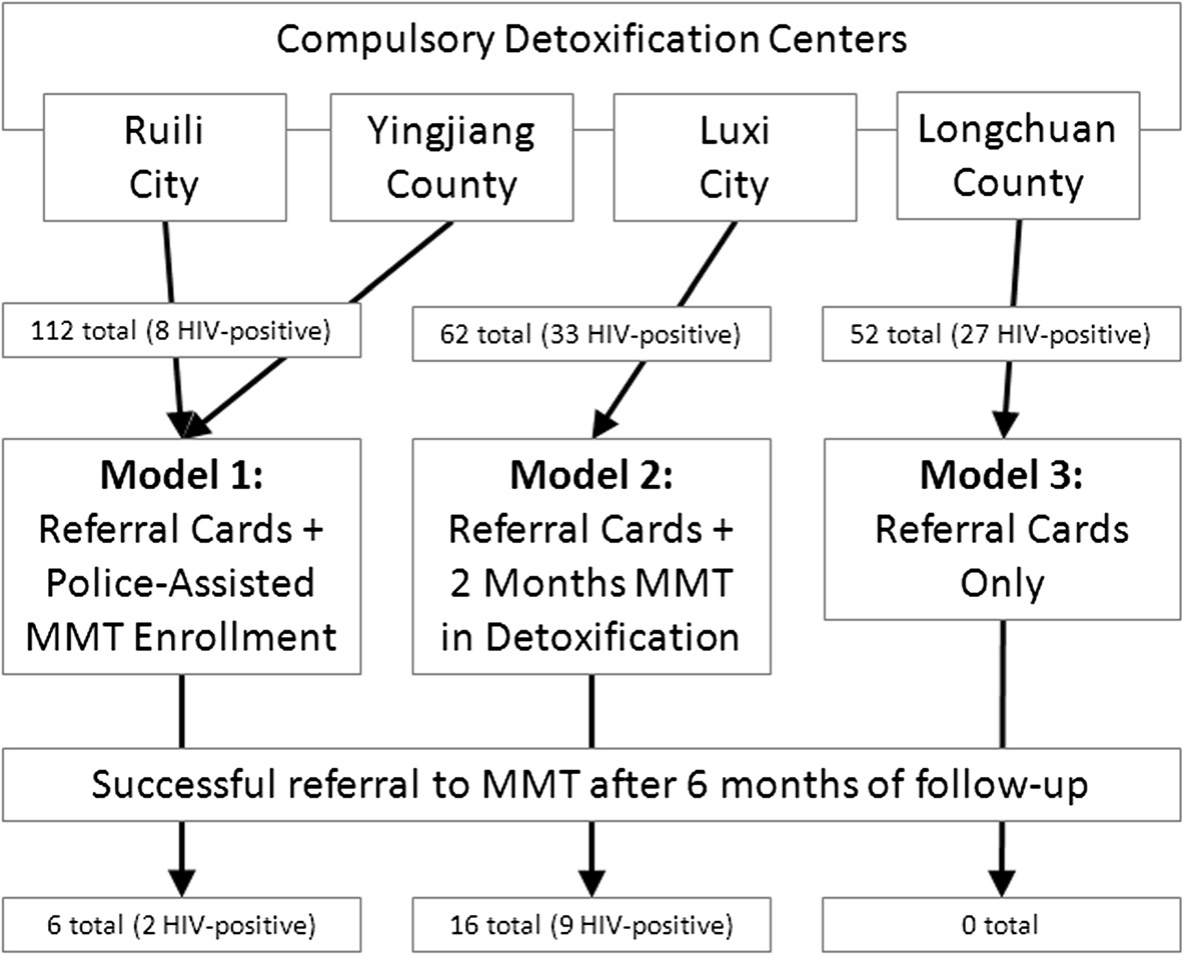
Schematic depicting the assignment of heroin addicts released from compulsory detoxification centers to one of the three referral strategy models examined in this study and the level of success observed in referring them to community MMT programs after six months of follow-up.

A quasi-experimental study design was used to test the effectiveness of three different methods for referring heroin addicts released from detoxification centers to community-based MMT programs. Four detoxification centers served as study sites—Luxi city, Ruili city, Longchuan county, and Yingjiang county, all in Dehong prefecture, Yunnan province, China. Study sites were selected based on existing relationships between the local government, police force, and Chinese Centers for Disease Control and Prevention (CDC) staff to maximize the effectiveness of each intervention model. Each of the four detoxification centers were assigned to one of three different referral models as described in Figure 1.

### Referral models

Model 1 was assigned to two detoxification centers, one in Ruili city and one in Yingjiang county. In this model, each subject was given one referral card and was escorted upon release from the detoxification center to an MMT clinic and enrolled in the MMT program by a police officer. Model 2 was assigned to the detoxification center in Luxi city. In this model, each subject was given MMT for two months in the detoxification center prior to release and then given a referral card upon release. Model 3 was assigned to the detoxification in Longchuan county and subjects assigned to this model were given a referral card only. In all cases, the referral card contained the contact information of a local MMT clinic and instructions on how to enroll as a patient.

### Study subjects

Study inclusion criteria required each participant to be [1] a registered heroin addict detained in one of the four selected detoxification centers, [2] scheduled for release in May 2009, [3] a legal citizen of the city/county where they were detained, and [4] more than 18 years old. A total of 226 participants met the inclusion criteria, participated in one of the three referral models, and were followed for six months, from the time of their release in May 2009, to the end of the study period in November 2009. A successful referral was defined as a referred heroin addict being treated in an MMT clinic for at least one month.

### Study survey

Prior to commencement of the study, local Center for Disease Control and Prevention (CDC) staff members completed two weeks of training on interviewing and data collection procedures. Two months prior to their scheduled release from the detoxification centers, all subjects participated in face-to-face interviews during which information was collected pertaining to their demographic characteristics, history of heroin use, prior participation in drug addiction-related interventional programs, and attitudes toward MMT programs.

### Sample collection, HIV testing, and counseling

Immediately following the completion of interviews (two months prior to release), venous blood samples (5 ml) were collected from all subjects by trained local CDC staff members. Blood collection, transportation, and laboratory processing, as well as testing quality assurance and control procedures were outlined in the study protocol. In brief, all blood samples were required to be transported within 12 hours of collection, all plasma samples were required to be extracted and stored at −20°C within 12 hours of arrival, and all tests were required to be conducted at laboratories certified by the National Center for AIDS/STD Control and Prevention (NCAIDS).

Blood samples were screened for HIV using a rapid test (Acon Biotech Co. Ltd, China) and positive samples were rescreened using a different rapid test (Standard Diagnostics, Inc., Korea). A positive result from both tests was considered to have been screened HIV-positive. All samples that either screened HIV-positive or yielded one positive and one negative outcome were retested using an ELISA (Kehua Biotech Co. Ltd., China) and samples positive for HIV via ELISA were re-tested by Western blot (MP Co. Ltd., China) and considered confirmed HIV-positive. All tests were performed according to the manufacturers’ instructions.

Counseling was provided onsite both before and after testing, and all subjects were informed of their HIV status prior to their release from the detoxification centers. Participants who tested HIV positive were referred to the National AIDS Program to assess their eligibility for anti-retroviral treatment.

### Statistical analysis

Baseline characteristics of the study subjects in the three intervention model groups were compared by χ^2^ test and Fisher’s exact test. To account for the site-related cluster effect of heroin addicts released from the same detoxification center, we conducted multilevel (detoxification center level and individual level) logistic regression analysis that included a random effect for each detoxification center to examine associations between referral models and the status of successful referral. Adjusted odds ratios (ORs) and 95% confidence intervals (CIs) obtained from the adjusted model including all covariates are reported. All p-values are two-sided and p < 0.05 was considered statistically significant. There were too few HIV-positive participants in the sample to conduct a multilevel logistic regression on this subgroup. Therefore, we used Fisher’s exact T-test to compare the number of referrals of HIV-positive subjects between intervention models. All statistical analyses were performed using SAS software (Version 9.2, SAS Institute, Cary, USA).

### Ethical approval

This study was reviewed and approved by the Institute Review Board of NCAIDS, Chinese CDC. Informed consent was obtained from all participants at the time of their enrollment in the study.

## Results

A total of 226 subjects participated in this study (Table 1). Most study participants were aged 18–34 years (64.2%), male (96.0%), Han Chinese (61.1%), and had junior high school-level educations or less (91.0%). Most participants reported having used heroin for six years or more (55.3%) and never having participated in an MMT program prior to detention in the detoxification center (85.0%). Additionally, most subjects had never participated in a needle-exchange program (97.3%) and a large proportion tested positive for HIV (30.1%).

**Table 1:** Selected characteristic of study participants overall and within each referral model

**Characteristics**	**Totals**	**Model 1: Referral cards + police assisted MMT enrollment**	**Model 2: Referral cards +2 months MMT in detoxification**	**Model 3: Referral cards only**	P-value^*^
	**N (%)**	**N (%)**	**N (%)**	**N (%)**	
Age (years)					
18 - 24	23 (10.2)	17 (15.2)	2 (3.2)	4 (7.7)	0.2237
25 - 34	122 (54.0)	57 (50.9)	34 (54.8)	31 (59.6)	
35 - 44	63 (27.9)	31 (27.7)	20 (27.7)	12 (23.1)	
≥ 45	18 (8.0)	7 (6.2)	6 (6.3)	5 (9.6)	
Gender					
Male	217 (96.0)	109 (97.3)	58 (93.5)	50 (96.2)	0.4018
Female	9 (4.0)	3 (2.7)	4 (6.5)	2 (3.8)	
Ethnicity					
Han	138 (61.1)	66 (58.9)	37 (59.7)	35 (67.3)	0.5719
Minority	88 (38.9)	46 (41.1)	25 (40.3)	17 (32.7)	
Education level					
< Primary school	121 (52.5)	56 (50.0)	28 (45.2)	37 (71.1)	0.0475
Junior high school	87 (38.5)	47 (42.0)	29 (46.8)	11 (21.2)	
≥Senior high school	18 (8.0)	9 (8.0)	5 (8.0)	4 (7.7)	
Marital status					
Married	95 (42.0)	43 (38.4)	28 (45.2)	24 (46.2)	0.7784
Divorced or widowed	29 (12.8)	16 (14.3)	6 (9.7)	7 (13.5)	
Never married	102 (45.1)	53 (47.3)	28 (45.2)	21 (40.4)	
HIV status					
Positive	68 (30.1)	8 (7.1)	33 (53.2)	27 (51.9)	<0.0001
Negative	158 (69.9)	104 (92.9)	29 (46.8)	25 (48.1)	
History of heroin use (years)					
< 1	30 (13.3)	16 (14.3)	8 (12.9)	6 (11.5)	0.0542
1-5	71 (31.4)	44 (39.3)	11 (17.7)	16 (30.8)	
6-10	57 (25.2)	28 (25.0)	17 (27.4)	12 (23.1)	
> 10	68 (30.1)	24 (21.4)	26 (41.9)	18 (34.6)	
Prior participation in a needle-exchange program					
Yes	6 (2.7)	2 (1.8)	1 (1.6)	3 (5.8)	0.3682
No	220 (97.3)	110 (98.2)	61 (98.4)	49 (94.2)	
Prior participation in an MMT program					
Yes	34 (15.0)	15 (13.4)	17 (27.4)	2 (3.8)	0.0017
No	192 (85.0)	97 (86.6)	45 (72.6)	50 (96.2)	
Willingness to participate in an MMT program					
Yes	116 (51.3)	48 (42.9)	44 (71.0)	24 (46.2)	0.0015
No	109 (48.2)	63 (56.3)	18 (29.0)	28 (53.8)	
Distance from home to the nearest MMT clinic (km)					
< 5	67 (29.6)	36 (32.1)	15 (24.2)	12 (23.1)	0.3619
5-10	41 (18.1)	23 (20.5)	11 (17.7)	7 (13.5)	
> 10	118 (52.2)	51 (45.5)	36 (58.1)	31 (59.6)	
Overall	226 (100)	112 (49.6)	62 (27.4)	52 (23.0)	

*P-values were calculated by χ^2^ and Fisher’s exact tests.

Of the 226 study participants, 112 were assigned to Model 1 (49.5%), 62 were assigned to Model 2 (27.4%), and 52 were assigned to Model 3 (23.0%). Upon comparison of the characteristics of these three subgroups, we found that a greater proportion of participants assigned to Model 3 had a primary school level education or less (71.1%, p < 0.0475), a lesser proportion of participants assigned to Model 1 had an HIV-positive serostatus (7.1%, p < 0.0001), and a greater proportion of participants assigned to Model 2 reported being willing to participate in an MMT program (71.0%). No other factors were found to differ significantly between the three subgroups (Table 1).

As shown in Table 2, a total of 22 participants (9.7%) were successfully referred to a community-based MMT program within six months of their release from detoxification centers. Six successful referrals (5.4%) were facilitated by Model 1, sixteen (25.8%) by Model 2, and zero by Model 3. The highest rate of successful referral was achieved with Model 2, subjects assigned to this model received MMT for a median of 83 days (mean = 87 days) during six months of follow-up.

**Table 2:** Comparisons of the three referral models during six months of follow-up

**Variables**	**Subjects**	**Subjects referred**	**Referral rate**	**Unadjusted**	**Adjusted**	**P-value**
	**N (%)**	**n (%)**	**(n/N,%)**	**OR (CI)**	**OR (CI)**	
*Referral strategy*						
**Model 1:** Referral Cards + Police-Assisted MMT Enrollment	112 (49.6)	6 (27.3)	5.4	1.0	1.0	
**Model 2:** Referral Cards + 2 Months MMT in Detoxification	62 (27.4)	16 (72.7)	25.8	1.2 (1.1-1.4)	1.2 (1.1-1.3)	0.0023
**Model 3:** Referral Cards Only	52 (23.0)	0 (0.0)	0.0	0.95 (0.83-1.1)	0.99 (0.90-1.1)	0.0757
*Baseline covariates*						
Age (years)						
18 - 24	23 (10.2)	0 (0.0)	0.0	1.0	1.0	-
25 - 34	122 (54.0)	13 (59.1)	10.7	0.97 (0.81-1.2)	1.0 (0.84-1.2)	0.9966
35 - 44	63 (27.9)	9 (40.9)	14.3	1.1 (0.97-1.3)	1.1 (0.96-1.3)	0.1662
≥ 45	18 (8.0)	0 (0.0)	0.0	0.97 (0.81-1.2)	1.0 (0.84-1.2)	0.9966
Gender						
Female	9 (4.0)	0 (0.0)	0.0	1.0	1.0	-
Male	217 (96.0)	22 (100)	10.1	1.2 (0.96-1.4)	0.96 (0.81-1.1)	0.6477
Ethnicity						
Minority	88 (38.9)	13 (59.1)	14.8	1.0	1.0	-
Han	138 (61.1)	9 (40.9)	6.5	0.93 (0.86-1.0)	0.96 (0.90-1.0)	0.2631
Education level						
< Primary school	121 (52.5)	5 (22.7)	4.1	1.0	1.0	-
Junior high school	87 (38.5)	13 (59.1)	14.9	1.1 (1.0-1.2)	1.0 (0.95-1.1)	0.6598
≥ Senior high school	18 (8.0)	4 (18.2)	22.2	1.2 (1.0-1.4)	1.1 (0.92-1.2)	0.4434
Marital status						
Never married	102 (45.1)	16 (72.7)	15.7	1.0	1.0	-
Married	95 (42.0)	5 (22.7)	5.3	0.90 (0.83-0.97)	0.94 (0.87-1.0)	0.1041
Divorced or widowed	29 (12.8)	1 (4.5)	3.4	0.89 (0.80-1.0)	0.91 (0.81-1.0)	0.0932
HIV status						
Negative	158 (69.9)	11 (50.0)	7.0	1.0	1.0	-
Positive	68 (30.1)	11 (50.0)	16.2	0.94 (0.86-1.0)	0.98 (0.90-1.1)	0.6770
History of heroin use (years)						
< 1	30 (13.3)	0 (0.0)	0.0	1.0	1.0	-
1-5	71 (31.4)	3 (13.6)	4.2	1.1 (1.0-1.3)	1.0 (0.93-1.2)	0.4783
6-10	57 (25.2)	9 (40.9)	15.8	1.2 (1.0-1.3)	1.0 (0.93-1.2)	0.4948
> 10	68 (30.1)	10 (45.5)	14.7	1.1 (1.0-1.3)	1.0 (0.93-1.2)	0.4783
Prior participation in a needle-exchange program						
No	220 (97.3)	21 (95.5)	9.5	1.0	1.0	-
Yes	6 (2.7)	1 (4.5)	16.7	1.1 (0.90-1.4)	1.2 (0.97-1.5)	0.0880
Prior participation in an MMT program			0.0			
No	192 (85.0)	5 (22.7)	2.6	1.0	1.0	-
Yes	34 (15.0)	17 (77.3)	50.0	1.6 (1.4-1.7)	1.5 (1.3-1.6)	<0.0001
Willingness to participate in an MMT program						
No	109 (48.2)	6 (27.3)	5.5	1.0	1.0	-
Yes	116 (51.3)	16 (72.7)	13.8	1.0 (0.97-1.1)	0.98 (0.92-1.1)	0.6527
Distance from home to the nearest MMT clinic (km)						
> 10	118 (52.2)	5 (22.7)	4.2	1.0	1.0	-
5-10	41 (18.1)	5 (22.7)	12.2	1.1 (0.98-1.1)	1.1 (0.96-1.1)	0.2565
< 5	67 (29.6)	12 (54.5)	17.9	1.2 (1.1-1.3)	1.0 (0.95-1.1)	0.4387
Overall	226 (100.0)	22 (100.0)	9.7	-	-	-

The results of multilevel logistic regression analysis are summarized in Table 2. Compared with Model 1, Model 2 was associated with increased odds of successful referral (adjusted OR = 1.2, CI = 1.1-1.3), and Model 3 did not different from Model 1. Prior MMT treatment was the only variable examined that was found to be associated with an increased likelihood of successful referral to MMT (adjusted OR = 1.5, CI = 1.3-1.6).

The overall rate of successful referral was greater among HIV-positive subjects (11 out of 68, 16.2%) compared to HIV-negative subjects (11 out of 158, 7.0%, Table 1). Comparison of successful referral rates among the 68 HIV-positive participants is shown in Table 3. The rate of successful referral to MMT was 25.0% for Model 1, 27.3% for Model 2, and 0% for Model 3.

**Table 3:** Comparisons of three referral models during six months of follow-up for HIV-positive subjects

**Referral strategy**	**HIV-Positive subjects**	**Subjects referred**	**Referral rate**	P-value^*^
	**N (%)**	**n (%)**	**(n/N,%)**	
**Model 1:** Referral Cards + Police-assisted MMT enrollment	8 (11.8)	2 (18.2)	25.0	0.0048
**Model 2:** Referral cards + 2 months MMT in detoxification	33 (48.5)	9 (81.8)	27.3	
**Model 3:** Referral cards only	27 (39.7)	0 (0)	0	
Overall	68 (100.0)	11 (100.0)	16.2	-

^*^Fischer’s exact t-test was used because of the small sample size.

## Discussion

A large proportion of China’s heroin addicts are incarcerated in compulsory detoxification centers where methadone treatment is unavailable [9]. Once released from these centers, relapse rates are estimated to be greater than 90% [6-9]. The probability of acquiring HIV is considered high in this population, due to high-risk, drug use-related injecting and sexual behaviors, both inside and outside of detoxification centers [9]. Expansion of methadone treatment coverage to include recently-released heroin addicts is critical to harm reduction in this population. However, effective interventions aimed at improving referral to community-based MMT clinics for newly-released prisoners in China are lacking.

This study evaluated the effectiveness of three referral models at successfully transitioning heroin users from compulsory detoxification to MMT participation. Findings show that use of a referral model that incorporates methadone treatment for heroin addicts while they are still incarcerated increases the probability of their enrollment and participation in MMT after their release. Adjusted analyses indicate that prior experience with methadone is the only study variable associated with increased odds of successful MMT referral. These results have important implications for both heroin addiction treatment and HIV transmission prevention in China, as they suggest that treating incarcerated heroin users with methadone prior to their release may promote their subsequent enrollment and participation in MMT. Additional research among heroin users from different regions in China is needed to further confirm the effectiveness of this referral card plus MMT intervention in increasing transition from a detoxification center to MMT enrollment and participation. A lager sample will be required to elucidate the severity (e.g., level of heroin use, HIV status) or enabling (e.g., perceived effectiveness of prior MMT or attitudinal changes after MMT) factors that may mediate or explain the association between prior MMT use and subsequent MMT participation.

Internationally, many studies have shown that out-of-treatment heroin addicts face many barriers to entry into MMT programs, including a long waiting list, lack of money or health insurance, beliefs about methadone’s real or rumored side effects, and fear of addiction to or later withdrawal from methadone [18-20]. Such barriers also are reported to exist for China’s opiate addicts [21]. Hence, interventions aimed at reducing or eliminating these barriers are urgently needed. Our results suggest that prior methadone treatment experience might help heroin users overcome some of these barriers, as demonstrated by the much higher proportion of successful referrals among heroin users in the referral card plus MMT while in detoxification intervention model (25.8%). However, China’s detoxification centers and prisons presently do not allow their detainees to receive methadone treatment despite the fact that both the World Health Organization and the United Nations Office on Drugs and Crime recommend use of opiate replacement treatment for heroin addicts in prison systems [22]. That said, China is not the only country that does not follow this guidance—at present only 29 countries, most of which are in Europe, provide opiate replacement therapy in their prison systems [22,23].

Unfortunately, heroin addicts in prison continue to be at high risk of acquiring blood-borne infections [24-27]. One study conducted in China has shown that injecting drug use behaviors are present among heroin addicts in detoxification centers [28]. Although it cannot be determined from the data whether HIV infections occurred prior to or during their terms in the detoxification centers, a relatively high proportion (30.1%) of participants in this study were HIV-positive. The high prevalence of HIV infection coupled with possible drug-related risk behaviors all point to a need for including addiction treatment (e.g., MMT) in detoxification centers. Of note, a higher proportion of HIV-positive heroin addicts (16.2%) were successfully referred to MMT as compared to HIV-negative subjects (7.0%) in our study. Although this result is descriptive and based on a small sample of HIV-positive participants (n = 68), it provides some evidence upon which future research studies can be based to test and refine referral models aiming to improve engagement in MMT after detoxification center release.

This study has several limitations. First, the sample size is small and the study sites are limited to Dehong prefecture. Therefore, the generalizability of this study may be limited. Second, we only examined whether heroin addicts successfully enrolled and participated in MMT. Some proportions of those who did not may have engaged in needle exchange programs or other treatment programs, such as buprenorphine treatment. Third, the intervention was not randomly assigned to detoxification centers, therefore the baseline characteristics of study subjects in the three intervention models were not balanced. Although our analyses adjusted for characteristics that differed across centers, we may not have been able to balance out all site-related differences. Thus, some selection bias may have remained. Fourth, there might some other important factors that could influence successful referral such as family supports were not collect by this study.

## Conclusion

In summary, these results provide important new evidence for the beneficial effect of prior methadone experience in promoting the successful MMT enrollment and participation by heroin addicts released from China’s compulsory detoxification centers. These data lend some support for establishing collaboration between China’s Ministry of Public Security and Ministry of Health to expand methadone treatment coverage such that detainees can be treated both during their sentences and after their release, which will not only prevent their likely relapse, but promote their rehabilitation, protect them from HIV infection, and increase their survival.

## Competing interest

The authors declare that they have no competing interests.

## Authors’ contributions

LY and ZW designed the study. LY, SD and ZW implemented the study. EL conducted data analysis. LY, EL, JMM, LW, SC, and ZW interpreted data and authored and edited the manuscript. All authors read and approved the final manuscript.

## Pre-publication history

The pre-publication history for this paper can be accessed here:

http://www.biomedcentral.com/1471-2458/13/747/prepub
